# A Hong Kong Chinese kindred with familial hypocalciuric hypercalcaemia caused by *AP2S1* mutation

**DOI:** 10.12688/f1000research.20344.1

**Published:** 2019-09-09

**Authors:** Felix Chi Kin Wong, Wai Sheung Wong, Jeffrey Sung Shing Kwok, Teresa Kam Chi Tsui, Kam Piu Lau, Michael Ho Ming Chan, Yuet Ping Yuen

**Affiliations:** 1 Department of Chemical Pathology, Prince of Wales Hospital, Shatin, Hong Kong; 2 Department of Medicine, North District Hospital, Sheung Shui, Hong Kong

**Keywords:** Familial hypocalciuric hypercalcaemia type III, AP2S1, Hong Kong Chinese

## Abstract

Familial hypocalciuric hypercalcaemia (FHH) is a genetic disorder of altered calcium homeostasis. Mutations in the *CASR*, *GNA11* and *AP2S1* genes have been reported to cause FHH. We report a Hong Kong Chinese kindred with FHH type 3 (FHH3) caused by mutations in *AP2S1*. The proband, a 51-year-old woman with hypercalcaemia, was initially diagnosed to have primary hyperparathyroidism but repeated parathyroidectomy failed to normalize her plasma calcium concentrations. Later, FHH was suspected and yet no mutations were identified in the *CASR* gene which causes FHH type 1 (FHH1), the most common form of FHH. Genetic testing of *AP2S1* revealed a heterozygous c.43C>T (p.Arg15Cys) mutation, confirming the diagnosis of FHH3. The elder brother and niece of the proband, who both have hypercalcaemia, were found to harbour the same mutation. To our knowledge, this is the first Chinese kindred of FHH3 reported in the English literature.

## Introduction

Familial hypocalciuric hypercalcaemia (FHH) is a genetically heterogeneous, autosomal dominant disorder characterized by a lifelong increase in plasma calcium concentrations with an inappropriately low urinary calcium excretion. In FHH, there is a reduction in the calcium-sensing ability of the chief cells of the parathyroid glands as well as an increase in tubular calcium reabsorption, resulting in an elevated homeostatic set-point of plasma calcium concentration and low urinary calcium excretion. Patients with FHH are generally asymptomatic, although some may develop pancreatitis or chondrocalcinosis ^1^. Inactivating mutations in *CASR* was first reported in 1993 to cause FHH1, the most common form of FHH ^2^. More recently, mutations in *GNA11* ^3^ and *AP2S1* ^4^ were identified to be responsible for FHH2 and FHH3, respectively. The following case report describes a Hong Kong Chinese kindred with hypercalcaemia and molecular diagnosis of FHH3. To our knowledge, this is the first Chinese kindred of FHH3 reported in the English literature.

## Case report

The proband was a 51-year-old Hong Kong Chinese woman who had an incidental finding of hypercalcaemia during hospitalization for *Vibrio parahaemolyticus* gastroenteritis. She presented with watery diarrhea, vomiting and colicky abdominal pain with dehydration. Results of laboratory tests on admission were as follows: plasma sodium 143 mmol/L (reference range [RR] 135 – 145 mmol/L), potassium 3.7 mmol/L (RR 3.5 – 5.1 mmol/L), creatinine 66 μmol/L (RR 44 – 80 μmol/L), urea 6.2 mmol/L (RR 2.7 – 6.8 mmol/L), total protein 83 g/L (RR 63 – 81 g/L) and albumin 49 g/L (RR 35 – 50 g/L). Unexpectedly, a grossly elevated plasma calcium level of 3.03 mmol/L (RR 2.10 – 2.55 mmol/L) was found. The plasma phosphate was 1.07 mmol/L (RR 0.90 – 1.55 mmol/L) and plasma alkaline phosphatase (ALP) was 97 U/L (RR 35 – 104 U/L). The patient had a past history of multi-nodular goiter and uterine fibroids with total hysterectomy and salpingo-oophorectomy performed in 1999. She did not have symptoms suggestive of hypercalcaemia, primary hyperparathyroidism or urolithiasis. She never took any calcium or vitamin supplements, and was not on any regular or over-the-counter medications.

After rehydration, the plasma calcium was persistently elevated at 2.79 mmol/L. Whole blood ionized calcium was elevated at 1.47 mmol/L (RR 1.13 – 1.32 mmol/L), confirming genuine hypercalcaemia. The paired plasma parathyroid hormone (PTH) level was inappropriately normal (6.9 pmol/L, RR 1.7 – 9.2 pmol/L). Urinary calcium excretion over 24 hours was normal at 3.26 mmol/day (RR 2.50 – 8.00 mmol/day) with a urine volume of 2.23 L. Spot urine calcium and creatinine concentrations were also measured one day before the aforementioned 24-hour urine collection with paired measurement of plasma calcium and creatinine concentrations. The fractional excretion of calcium (FECa) was not documented in the patient’s medical record. In retrospect, it was low (0.55%, see Table 1, six months after presentation). Blood for serum protein electrophoresis, immunoglobulin pattern, erythrocyte sedimentation rate (ESR), and chest X-ray were unremarkable. Ultrasonography showed multi-nodular disease of thyroid gland and no parathyroid mass. Technetium-99m sestamibi parathyroid scintigraphy showed a small extra-thyroidal uptake focus near the lower pole of the right thyroid lobe suggestive of a right inferior parathyroid adenoma. A diagnosis of primary hyperparathyroidism due to right inferior parathyroid adenoma was made.

**Table 1.** Summary of the biochemical findings of all affected family members. Biochemical results measured on the same day are tabulated on the same row. The FECa of all affected family members was consistently below 1% ( **bold**). *Age at which the patient first presented with hypercalcaemia. ^†^Calculation of the fractional excretion of calcium (FECa) is as follows: ([urine calcium] × [plasma creatinine])/([urine creatinine] × [plasma calcium]) × 100%. ^‡^During the episode of acute pancreatitis. Reference ranges: plasma calcium (2.10 – 2.55 mmol/L), plasma phosphate (0.90 – 1.55 mmol/L), plasma creatinine (44 – 80 μmol/L), plasma PTH (1.7 – 9.2 pmol/L). Abbreviations: Ca, calcium; Cr, creatinine; FECa, fractional excretion of calcium; PO _4_, phosphate.

Patient No.	Age (years) *	Time of measurement	Plasma Ca (mmol/L)	Plasma PO _4_ (mmol/L)	Plasma Cr (umol/L)	Type of urine specimen	Urine Ca (mmol/L)	Urine Cr (mmol/L)	FECa (%) ^†^	Plasma PTH (pmol/L)
II(4) (proband)	51	At presentation	3.03	1.07	66	--	--	--	--	6.9
		Six months later	2.84	1.01	58	Spot	0.91	3.4	**0.55**	--
		2 years later	2.63	1.10	56	24 hour	0.62	1.9	**0.69**	--
II(1)	54	At presentation	2.80	0.71	57	--	--	--	--	3.2
		5 years later	2.89	0.94	68	Spot	4.43	12.6	**0.83**	--
III(1)	29	At presentation	2.69	0.40	54	--	--	--	--	5.1
		2 years later ^‡^	2.65	0.95	42	--	--	--	--	1.5
		2 years and 2 days later ^‡^	2.69	0.99	39	Spot	0.3	4.9	**0.09**	--
		3 years later	2.66	0.92	61	Spot	0.9	11.5	**0.15**	--
		7 years later	2.60	--	50	Spot	3.47	8.9	**0.75**	--

Right inferior parathyroidectomy was performed 2 years after initial presentation. A 1 × 0.5 × 0.3 cm ovoid nodule was excised and frozen section showed a piece of parathyroid tissue without evidence of malignancy. However, the patient had persistent hypercalcaemia postoperatively with a plasma PTH level of 5.5 pmol/L. In addition, the surgical site was complicated by local haematoma formation. A second operation for haemostasis and subtotal parathyroidectomy was performed three days after the first operation. In order to maximize the probability of excising any hyperactive parathyroid tissue so that the normalization of plasma calcium level may be achieved, the right superior parathyroid gland and left inferior parathyroid gland were also excised, leaving only the left superior parathyroid gland in place. Histological examination confirmed further removal of parathyroid tissue with no evidence of parathyroid neoplasm. Nevertheless, her plasma calcium level remained elevated at levels of 2.63 to 2.84 mmol/L while the plasma PTH level remained non-suppressed at 4.6 pmol/L after the second operation. Technetium-99m sestamibi parathyroid scintigraphy three months after the second operation showed a suspicious right inferior hyperfunctioning parathyroid lesion, raising the possibility of residual parathyroid disease.

**Figure 1.**
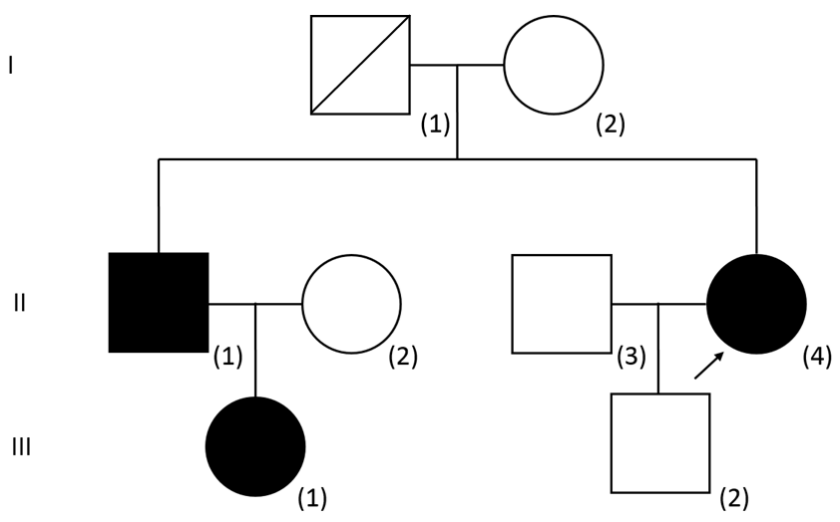
The pedigree of this family was compatible with autosomal dominant inheritance of hypercalcaemia. The proband is indicated by an arrow. The phenotype (plasma calcium concentration) as well as genotype of both parents of the proband is unknown. The plasma calcium concentration of the son of the proband [III(2)] was normal and targeted *AP2S1* mutation analysis was negative. The calcium status of the baby boy of III(1) was not available and it was omitted from the pedigree.

After failing parathyroidectomy twice, the patient realized for the first time that her elder brother and her elder brother’s daughter also had incidental findings of hypercalcaemia (See Figure 1 for the pedigree and Table 1 for the summary of biochemical results). Upon further evaluation of the proband, 24-hour urine collection was repeated revealing a low urinary calcium excretion [2.09 mmol/day, urine volume 3.37 L, (RR 2.50 – 8.00 mmol/day)]. Spot urine calcium was 0.62 mmol/L and spot urine creatinine was 1.9 mmol/L. The concomitant plasma calcium was 2.63 mmol/l and plasma creatinine was 56 μmol/L. The FECa was 0.69%. A FECa of less than 1% is compatible with a diagnosis of FHH.

The elder brother of the proband presented at the age of 54 years with an incidental finding of hypercalcaemia (plasma calcium: 2.80 mmol/L) during hospitalization for an episode of syncope. Measurement of 24-hour urinary calcium excretion was not performed. FECa was 0.83%. The niece of the proband presented at the age of 29 years with an incidental finding of hypercalcaemia (plasma calcium: 2.69 mmol/L) during hospitalization for urinary tract infection. Two years later, she had an episode of acute pancreatitis during pregnancy at 37 weeks’ gestation with serum amylase up to 1377 U/L. The concomitant triglyceride level was 1.7 mmol/L (RR <1.7 mmol/L) and plasma calcium was 2.65 – 2.69 mmol/L. Emergency Caesarean section was performed with uneventful delivery of a healthy baby boy, although the calcium status of her baby was unknown. Her pancreatitis responded to conservative management. Ultrasound of the neck showed no parathyroid nodules. Urinary calcium excretion over 24 hours was measured on two occasions but both results were within normal limits (4.92 and 3.18 mmol/day collected three years apart, with urine volumes of 3.3 and 3.5 L/day respectively). FECa was persistently low (0.09 – 0.75%).

Mutation analysis of the calcium-sensing receptor ( *CASR*) gene was performed for the proband with all coding exons and flanking introns sequenced in both directions. No known pathogenic variants were identified. As a result, a diagnosis of FHH type 1 could not be confirmed. Sanger sequencing of the adaptor-related protein complex 2, sigma-1 subunit ( *AP2S1*) gene showed heterozygous *AP2S1* NM_004069.3: c.43C>T (p.Arg15Cys), which is a known pathogenic mutation of FHH3. The same *AP2S1* mutation was identified in both her elder brother and niece, confirming the diagnosis of FHH3 in all three individuals. All three patients appeared to be cognitively normal. They were followed up for the monitoring of plasma calcium concentration. Up to one year after the genetic diagnosis was made, they remained asymptomatic and no treatment was given.

## Discussion

FHH is an important differential diagnosis of hypercalcaemia that one must carefully differentiate from primary hyperparathyroidism. The most indicative biochemical parameter for the diagnosis of FHH is the fractional excretion of calcium (FECa), also known as urinary calcium to creatinine clearance ratio. It is typically less than 1% in patients with FHH ^5– 7^. As exemplified in this case report, the diagnosis of FHH could be missed if 24-hour urinary calcium excretion only is taken into consideration. For calculation of the FECa, a spot urine sample or a 24-hour urine sample may be used, although 24-hour urine samples were originally employed to derive the cut offs ^5, 6^. Plasma PTH levels may be normal or raised in patients with FHH, similar to patients with primary hyperparathyroidism. Frequently, it remains difficult to distinguish primary hyperparathyroidism and FHH based on the available clinical, biochemical and radiological evidence, and the definitive diagnosis of FHH could only be achieved by genetic testing. Approximately 65% of individuals with FHH has FHH1, which is the most common type of FHH due to a loss-of-function of the calcium-sensing receptor (CaSR) ^8^. More recently, mutations in *GNA11* and *AP2S1* have been identified to cause FHH2 and FHH3, respectively. FHH3 is caused by mutations at the Arg15 residue of the AP2S1 protein. Three different amino acid substitutions have been identified at this arginine residue, namely p.Arg15Cys (CGC➙TGC), p.Arg15Leu (CGC➙CTC) and p.Arg15His (CGC➙CAC). The Arg15 residue is highly evolutionarily conserved. *In vitro* functional studies suggest that a replacement of the positively charged Arg15 residue with the polar, but uncharged, Cys15 residue compromises the function of the adaptor-related protein complex 2 by reducing its affinity for the C-terminal calcium sensing receptor (CaSR) dileucine motifs, affecting the sensitivity of CaSR-expressing cells to extracellular calcium as well as resulting in the reduction of CaSR endocytosis ^4^. In the same study, 11 individuals were found to harbour FHH3 mutations in 50 unrelated individuals with *CASR* mutation-negative FHH. Therefore, it is likely that *AP2S1* mutations may be found in approximately 20% of patients of FHH without *CASR* mutations ^4^. FHH2 is much rarer than FHH1 or FHH3. In fact, not a single case of FHH2 was detected in studies which collectively involved more than 200 patients with a phenotype of FHH ^9– 11^. Compared with patients with FHH1, patients with FHH3 are more likely to have higher serum calcium and magnesium, and lower fractional excretion of calcium. In addition, cognitive dysfunction, such as learning disability and attention deficit hyperactivity disorder, was seen in some patients with FHH3 and this was not seen in patients with FHH1 ^11, 12^. No apparent cognitive dysfunction was seen in any of our patients, and magnesium levels were not checked. Cinacalcet, a licensed calcium-sensing receptor allosteric activator, has been used successfully to reduce plasma calcium levels and hypercalcaemic symptoms in symptomatic FHH3 patients ^13^. One of our patients ( Figure 1, [III(1)]) developed an episode of acute pancreatitis during pregnancy, with no recurrence afterwards. Otherwise, they were free of hypercalcaemic complications or symptoms, and therefore no treatment was given.

In summary, we have reported the first Chinese kindred with FHH3 in the English literature. Mutation analysis of *AP2S1* should be performed when the diagnosis of FHH is highly likely and yet no mutations in the *CASR* gene could be identified.

## Consent

Written informed consent for publication of their clinical details was obtained from all three patients.

## Data availability

### Underlying data

All data underlying the results are available as part of the article and no additional source data are required.
