# Supplementary material for: A Hong Kong Chinese kindred with familial hypocalciuric hypercalcaemia caused by AP2S1 mutation
Source: F1000Res. 2019 Sep 9;8:1612. [Version 1] doi: 10.12688/f1000research.20344.1 (PMC6826774; doi:10.12688/f1000research.20344.1)
Supplement: Peer review history

# Peer review: Reviewer response for version 1

**DOI:** 10.5256/f1000research.22357.r55718

**Published:** 2019-11-01

**Authors:** Pascal Houillier

**Affiliations:** 1 Department of Physiology, Assistance Publique Hôpitaux de Paris, Hôpital Européen Georges-Pompidou, Paris, France; 2 Paris Descartes University, Paris, France

Wong *et al *describe a Chinese kindred with FHH 3 due to a monoallelic point mutation in the AP2S1 gene. The phenotype of the proband and affected relatives is well described and the report is well written. I have a few comments that I submit to the consideration of the authors:

- The work-up of patients with primary hyperparathyroidism commonly includes renal ultrasonography (or CT scan) and bone mineral density measurement. Was any of those performed in any of the patients? If so, the results should be provided. If not, this should be mentioned.
- A score, named Pro-FHH, ^1^ has recently been reported as performing better that CCCR to distinguish patients with primary hyperparathyroidism and with FHH. Could Pro-FHH be retrospectively be computed in the patients? The respective merits of CCCR and Pro-FHH could be discussed.
- The description of the parathyroid tissue is a bit confusing. Is neoplasm used instead of carcinoma? Was the removed parathyroid tissue totally normal (on a quantitative and qualitative basis), or hyperplastic or anything?
- Table 1 should be cited when describing the biochemistry in the brother and the niece.
- Hospitalization for urinary tract infection is uncommon unless it is complicated. Was it?
-  The risk of post surgical hypoparathyroidism after repeated parathyroid surgery should be mentioned in the discussion.
- page 1, introduction: renal tubular calcium reabsorption

I confirm that I have read this submission and believe that I have an appropriate level of expertise to confirm that it is of an acceptable scientific standard, however I have significant reservations, as outlined above.

## References

[1] : Pro-FHH: A Risk Equation to Facilitate the Diagnosis of Parathyroid-Related Hypercalcemia. *The Journal of Clinical Endocrinology & Metabolism*.2018;103(7) : 10.1210/jc.2017-02773 2534-2542 10.1210/jc.2017-02773 29727008

# Peer review: Reviewer response for version 1

**DOI:** 10.5256/f1000research.22357.r54582

**Published:** 2019-10-21

**Authors:** Fadil M. Hannan

**Affiliations:** 1 Academic Endocrine Unit, Oxford Centre for Diabetes, Endocrinology and Metabolism, Radcliffe Department of Medicine, University of Oxford, Oxford, UK

The Clinical Practice Article by Wong and colleagues describes the first report of a Chinese kindred with familial hypocalciuric hypercalcaemia type 3 (FHH3). It is informative and well written, and provides detailed phenotypic information for this kindred. I have a few comments, which require addressing:

**Title:**

- Please insert details of the mutation e.g. “…hypercalcaemia caused by an AP2S1 mutation, Arg15Cys”

**Page 3: **

- In the paragraph detailing parathyroid histology in the proband, is the term 'without evidence of malignancy', referring to parathyroid adenoma or carcinoma? Malignancy should not be used interchangeably with parathyroid adenoma, given that this is a benign condition.
- Please avoid the term 'parathyroid neoplasm' as this suggests that histology is being undertaken to assess for parathyroid carcinoma.
- Please cite Table 1 in the paragraph describing the findings in the elder brother.
- Table 1 should be cited after the statement 'FECa was 0.83%'.
- Also cite Table 1 when describing the niece's biochemistry.
- Please state if gallstones were excluded as a cause of the acute pancreatitis

**Page 4:**

- This article would benefit from a more in depth discussion of the pancreatitis in the niece. The authors should mention that pancreatitis has previously been reported in a child with FHH3 (Scheers et al Pancreatology 2019). This reported patient also harboured a mutation in the SPINK1 gene, which represents a risk factor for pancreatitis. The authors should indicate whether or not the niece has been tested for a mutation in the SPINK1 gene.

**Table 1:**

- Please insert serum 25-hydroxyvitamin D values for members of this kindred.

**Figure 1: **

- Please insert sequence trace for the proband showing the c.43C>T nucleotide substitution in the AP2S1 gene.

I confirm that I have read this submission and believe that I have an appropriate level of expertise to confirm that it is of an acceptable scientific standard, however I have significant reservations, as outlined above.

# Peer review: Reviewer response for version 1

**DOI:** 10.5256/f1000research.22357.r54581

**Published:** 2019-10-16

**Authors:** Caroline Gorvin

**Affiliations:** 1 Institute of Metabolism and Systems Research, University of Birmingham, Birmingham, UK

Wong *et al* describe a Chinese kindred with FHH in which they have identified a previously described mutation in *AP2S1*. This is a comprehensive case report with detailed descriptions of the proband and several family members. Whilst not novel, this report describes FHH3 in a new ethnic population and further demonstrates that mutations in the Arg15 residue are the most commonly identified in FHH3. I have recommended some minor edits to improve the clarity of the report that are outlined below.  

  

1. At present there are very few references in the introduction. Perhaps a few more could be inserted.
2. Please add an asterisk (or other appropriate symbol) next to statistically significant values in Table 1. This will make it immediately clear to the reader which values are significant.
3. The statement about FECa being <1% in FHH is made on page 3. This should be brought forward to page 2 following the first statement about the proband’s FeCa.
4. The second paragraph of page 2 begins with a statement about other members of the proband’s family, then goes on to discuss the proband again. I think this statement should be moved down to just before the mutational analysis. It will make the case report easier for a reader to understand if the proband is discussed first, then other family members later.

I confirm that I have read this submission and believe that I have an appropriate level of expertise to confirm that it is of an acceptable scientific standard.
